# Long-term survival outcomes from a phase II trial of perioperative capecitabine plus oxaliplatin for advanced gastric cancer with extensive lymph node metastases: OGSG 1701

**DOI:** 10.1007/s10120-026-01726-x

**Published:** 2026-03-13

**Authors:** Shunji Endo, Yutaka Kimura, Naotoshi Sugimoto, Ryohei Kawabata, Atsushi Takeno, Shigeyuki Tamura, Jin Matsuyama, Masato Nakamura, Hiroki Takeshita, Motohiro Imano, Atsushi Yasuda, Hironaga Satake, Shogen Boku, Masahito Kotaka, Tomohira Takeoka, Yukinori Kurokawa, Toshimasa Tsujinaka, Toshio Shimokawa, Taroh Satoh

**Affiliations:** 1 Department of Digestive Surgery, Kawasaki Medical School, Kurashiki, Japan; 2 Department of Surgery, Yao Municipal Hospital, Yao, Japan; 3 Department of Gastroenterological Surgery, Higashiosaka City Medical Center, Higashiosaka, Japan; 4 Department of Surgery, Kindai University Nara Hospital, 1248-1 Otoda-cho, Ikoma, Nara, 630-0293 Japan; 5 Department of Surgery, Kindai University Faculty of Medicine, Sakai, Japan; 6 Department of Genetic Oncology, Osaka International Cancer Institute, Osaka, Japan; 7 Department of Surgery, Sakai City Medical Center, Sakai, Japan; 8 Department of Surgery, Osaka Rosai Hospital, Sakai, Japan; 9 Department of Surgery, Kansai Rosai Hospital, Amagasaki, Japan; 10 Department of Medical Oncology, Jisenkai Medical Corporation Aizawa Hospital, Matsumoto, Japan; 11 Department of Surgery, Matsushita Memorial Hospital, Moriguchi, Japan; 12 Department of Clinical Oncology, Kansai Medical University Hospital, Hirakata, Japan; 13 Gastrointestinal Cancer Center, Sano Hospital, Kobe, Japan; 14 Department of Gastroenterological Surgery, Hyogo Prefectural Nishinomiya Hospital, Nishinomiya, Japan; 15 Department of Gastroenterological Surgery, The University of Osaka Graduate School of Medicine, Suita, Japan; 16 Izumi City General Hospital, Izumi, Japan; 17 Department of Biostatistics, Faculty of Medicine, Wakayama Medical University, Wakayama, Japan; 18 Center for Cancer Genomics and Precision Medicine, The University of Osaka Hospital, Suita, Japan

**Keywords:** Stomach neoplasms, Neoadjuvant therapy, Lymphatic metastasis, Capecitabine, Oxaliplatin

## Abstract

**Background:**

Patients with advanced gastric cancer and para-aortic and/or bulky lymph node metastases have extremely poor prognosis after surgery alone. The OGSG1701 phase II trial evaluated the efficacy and safety of perioperative administration of capecitabine and oxaliplatin (CapeOx). Short-term results showed promising response and resection rates. Herein, we report the final survival outcomes.

**Methods:**

This multicenter, single-arm, phase II trial enrolled patients with histologically proven, HER2-negative or unknown gastric cancer with para-aortic (no.16a2/16b1) and/or bulky lymph node metastases. Patients received three cycles of preoperative CapeOx followed by gastrectomy with D2 ± para-aortic lymphadenectomy, and five cycles of postoperative CapeOx. The primary endpoint was response rate; the key secondary endpoints included overall survival (OS) and progression-free survival (PFS).

**Results:**

Thirty patients from 14 institutions were enrolled between 2018 and 2022. At a minimum follow-up of 36 months, the 3- and 5-years OS rates were 79.0% (95% confidence interval [CI], 59.0–90.0%) and 61.0% (95% CI 38.9–77.1%), respectively. Median OS was 64.9 months (95% CI 41.2–not estimable). The 3 years PFS rate was 46.7% (95% CI 28.4–63.0%), and the median PFS was 29.0 months (95% CI 9.4–not estimable). No additional treatment-related deaths occurred during the long-term follow-up.

**Conclusions:**

Perioperative CapeOx therapy resulted in encouraging long-term survival in patients with advanced gastric cancer and extensive nodal metastasis. These findings support its potential role as a perioperative strategy for biologically high-risk gastric cancer, with an acceptable safety profile.

**Supplementary Information:**

The online version contains supplementary material available at 10.1007/s10120-026-01726-x.

## Introduction

Gastric cancer is one of the leading causes of cancer-related mortality worldwide. Curative resection is essential for long-term survival and requires complete removal of the primary tumor and the involved regional lymph nodes (LNs). However, patients with extensive nodal disease, such as para-aortic lymph node (PALN) metastasis or bulky nodal involvement along the celiac axis and its branches, have extremely poor outcomes even after macroscopically complete resection. Extended lymphadenectomy, which often requires pancreatosplenectomy or other multivisceral resections, increases postoperative morbidity without providing sufficient survival benefits, highlighting the need for more effective systemic treatment strategies.

Several Japanese phase II trials evaluated the efficacy of preoperative chemotherapy for gastric cancer with extensive nodal involvement. In JCOG0001, two cycles of irinotecan plus cisplatin achieved a 55% response rate and a 3-year survival rate of 27%; however, treatment-related mortality reached 5%, limiting its feasibility in clinical practice [1]. In contrast, JCOG0405 demonstrated that two cycles of S-1 plus cisplatin (SP) produced a 65% response rate and favorable 3- and 5-year overall survival (OS) rates of 59% and 53%, respectively, without treatment-related deaths, establishing SP as a reference regimen in this setting [2]. The COMPASS trial assessed several combinations including S-1 plus cisplatin and paclitaxel plus cisplatin, although their efficacy remained modest [3]. JCOG1002 subsequently investigated docetaxel, cisplatin, and S-1 (DCS) followed by D2 plus PALN dissection and reported a clinical response rate of 57.7%; however, the primary endpoint was not met [4], and the 5-year OS rate of 54.9% did not clearly exceed that achieved with S-1 plus cisplatin in JCOG0405 despite greater treatment intensity and toxicity [5].

Postoperative adjuvant chemotherapy with capecitabine and oxaliplatin (CapeOx) is one of the standard regimens after D2 gastrectomy for stage II–III disease in Japan and Korea [6]. However, its benefit in patients with PALN or bulky LN metastases remains uncertain, and postoperative morbidity often compromises treatment compliance. These limitations support a perioperative strategy, in which chemotherapy begins preoperatively to enhance resectability, eradicate micrometastases, and ensure adequate systemic therapy. Dividing the standard eight cycles of adjuvant CapeOx into three preoperative and five postoperative cycles was expected to improve the treatment intensity while maintaining feasibility.

Therefore, the OGSG1701 trial prospectively evaluated the efficacy and safety of perioperative CapeOx in patients with gastric cancer and extensive LN metastasis. Short-term results showed a response rate of 66.7% and an R0 resection rate of 93.3% with acceptable toxicity [7]. Here, we report the final survival outcomes of patients treated with OGSG1701.

### Patients and methods

#### Study design and patients

The OGSG1701 trial was a prospective, multicenter, single-arm, phase II study conducted at 14 institutions in Japan. The trial adhered to the principles of the Declaration of Helsinki. The protocol was approved by the institutional review boards at all participating hospitals and was registered in the University Hospital Medical Information Network (UMIN000028749) and the Japan Registry of Clinical Trials (jRCTs051180186). Written informed consent was obtained from all patients before enrollment.

Eligible patients met the following criteria: (1) histologically confirmed gastric cancer; (2) Human epidermal growth factor receptor type 2 (HER2) -negative or unknown; (3) LN metastasis (> 1 cm based on enhanced computed tomography [CT]) in PALN stations (no. 16a2 or 16b1), or bulky LN metastasis (≥ 3 cm, or at least two adjacent tumors ≥ 1.5 cm) in stations no. 7, 8a, 9, 11, 12a, or 14v; (4) no mediastinal LN, pulmonary, peritoneal, liver, or other distant metastases on enhanced CT, no PALN metastasis other than no. 16a2/16b1, no pleural effusion or ascites; (5) no type 4 or type 3 tumor > 8 cm; (6) no esophageal invasion or invasion ≤ 3 cm; (7) no remnant gastric cancer; (8) no clinical signs of cervical LN or distant metastases; (9) age 20–80 years; (10) Eastern Cooperative Oncology Group performance status 0–1; (11) no prior chemotherapy, radiotherapy, or endocrine therapy for any malignancies; (12) no prior gastrectomy except bypass surgery and endoscopic resection; (13) fair oral intake with or without bypass surgery; (14) adequate organ function; and (15) written informed consent. Patients with synchronous or metachronous (within five years) malignancies other than carcinoma in situ or mucosal carcinoma, poorly controlled comorbidities, or severe medical conditions were excluded. Tumor staging and D classification were in accordance with the Japanese Classification of Gastric Carcinoma, third English edition, and seventh edition of the International Union against Cancer TNM staging system [8, 9].

#### Treatment

Preoperative CapeOx consisted of oral capecitabine 1000 mg/m^2^ twice daily (days 1–14) plus intravenous oxaliplatin 130 mg/m^2^ (day 1), repeated every 3 weeks. Three preoperative cycles were planned unless disease progression or unacceptable toxicity occurred. The tumor response was assessed after three cycles.

Surgery, including gastrectomy with D2 lymphadenectomy plus sampling of suspected nodes or PALN (no.16a2/16b1) dissection, was scheduled after preoperative chemotherapy. Concomitant resection of directly invaded organs was permitted if technically feasible; however, extended procedures such as left upper abdominal exenteration, pancreaticoduodenectomy, Appleby’s operation, peritonectomy, or total gastrectomy with subtotal esophagectomy were not allowed. If the surgical team deemed R0 resection impossible, treatment was discontinued.

Patients who underwent R0 resection received five postoperative cycles of CapeOx. In total, eight perioperative cycles were planned. At the investigator’s discretion, patients could undergo surgery after two preoperative cycles, followed by six postoperative cycles.

#### Endpoints

The primary endpoint was the response rate to preoperative therapy, centrally reviewed according to Response Evaluation Criteria in Solid Tumors (RECIST) v1.0 [10]. Secondary endpoints included 3-year OS, progression-free survival (PFS), R0 resection rate, protocol treatment completion, relative dose intensity (RDI) of capecitabine and oxaliplatin, pathological response (≥ grade 1b, Japanese Classification of Gastric Carcinoma), adverse events (AEs), and surgical complications. The response rate was also evaluated using RECIST v1.1 [11]. AEs were graded according to the Common Terminology Criteria for Adverse Events (CTCAE) v4.0 [12]. Surgical complications were classified using the Clavien–Dindo system [13].

#### Follow-up

All patients were followed for at least 3 years from registration, in accordance with standard practice at each participating institution. Imaging, including contrast-enhanced CT, was performed at regular intervals, typically every six months for the first three years and annually thereafter. Disease recurrence was confirmed using appropriate imaging modalities such as CT, ultrasonography, or endoscopy.

#### Statistical analysis

The expected response rate was set to 65% with reference to JCOG0405 [2], and the threshold response rate was set to 50%. Based on this design, 28 patients were required to ensure that the 90% confidence interval (CI) width was < 15% (one-sided), with a target accrual of 30 patients to allow for ineligible cases.

Clopper–Pearson’s exact method was used to calculate 90% CIs for the response rate. OS and PFS were estimated using the Kaplan–Meier method, and CIs were calculated using the Greenwood formula. Binary outcomes were summarized as proportions with exact 95% CIs. Statistical analyses were performed using R version 4.3.0 (R Foundation for Statistical Computing, Vienna, Austria).

## Results

### Patient characteristics and short-term outcomes

**Fig. 1:**
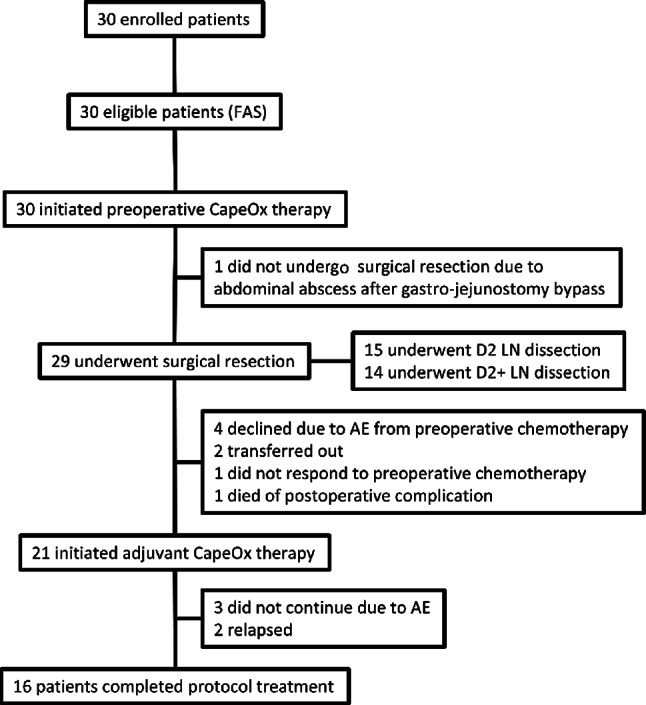
CONSORT diagram. *FAS* full analysis set, *CapeOx* capecitabine plus oxaliplatin, *LN* lymph node, *AE* adverse event

Thirty patients from 14 institutions were enrolled between March 2018 and June 2022 (Fig. 1). The baseline characteristics are summarized in Table 1. Half of the patients had bulky LN metastases, 56.7% had PALN metastases, and 6.7% had both types of metastases. Twenty-nine patients (96.7%) completed preoperative CapeOx; 28 completed all three cycles and 1 completed two cycles. The response rate according to RECIST v1.0, the primary endpoint, was 66.7% (90% CI 50.1–80.7; 95% CI 47.2–82.7), meeting the prespecified statistical threshold. The disease-control rate was 93.3% (95% CI 77.9–99.2). Gastrectomy was performed in 29 patients: distal gastrectomy in 15 patients (52%), total gastrectomy in 13 patients (45%), and proximal gastrectomy in 1 patient (3%). Lymphadenectomy included D2 dissection in 15 patients (52%) and D2 + in 14 patients (48%). The pathological findings of the resected patients are summarized in Table 2. The minor pathological response rate (grade ≥ 1b) was 66.7% (95% CI 47.2–82.7), and pathological complete response was achieved in 20.0% (95% CI 7.7–38.6). R0 resection was accomplished in 93.3% (95% CI 77.9–99.2). Postoperative CapeOx was initiated in 21 patients (70.0%; 95% CI 49.9–86.3) and completed in 16 (53.3% of the total cohort; 95% CI 34.3–71.7). The mean RDIs during preoperative therapy were 97.0% for capecitabine and 97.4% for oxaliplatin and 88.5% and 72.1% during postoperative therapy, respectively. The treatment-related toxicities were generally manageable, and no chemotherapy-related deaths occurred. One patient died due to postoperative complications. The detailed short-term outcomes have been reported previously [7] .

**Table 1:** Patient characteristics

	n = 30
Age, years	
Median (range)	66 (40–78)
Sex	
Male	24 (80%)
Female	6 (20%)
PS	
0	25 (83%)
1	5 (17%)
Main tumor location	
Upper third	12 (40%)
Middle third	8 (27%)
Lower third	9 (30%)
Esophagus	1 (3%)
Esophageal involvement	
Absent	26 (87%)
Present	4 (13%)
Macroscopic type	
0	3 (10%)
1	1 (3%)
2	9 (30%)
3	16 (53%)
5	1 (3%)
Histological type	
Differentiated	14 (47%)
Undifferentiated	16 (53%)
Depth of tumor invasion (T)	
cT2 (MP)	5 (17%)
cT3 (SS)	12 (40%)
cT4a (SE)	13 (43%)
Lymph node metastasis (N)	
cN1	10 (33%)
cN2	12 (40%)
cN3a	8 (27%)
Distant metastasis (M)	
cM0	13 (43%)
cM1	17 (57%)
cStage	
cStage IIB	4 (13%)
cStage IIIA	1 (3%)
cStage IIIB	7 (23%)
cStage IIIC	1 (3%)
cStage IV	17 (57%)
Node status	
Bulky lymph nodes only	13 (43%)
PALN only	15 (50%)
PALN and bulky lymph nodes	2 (7%)

*PS* Eastern Cooperative Oncology Group Performance Status, *PALN* para-aortic lymph node. Clinical findings of gastric cancer are documented according to the Japanese Classification of Gastric Carcinoma, third English edition

**Table 2:** Pathological findings for the resected patients

	n = 29
Depth of tumor invasion (T)	
ypT0	6 (21%)
ypT1a	1 (3%)
ypT1b	2 (7%)
ypT2	4 (14%)
ypT3	10 (34%)
ypT4a	6 (21%)
Lymph node metastasis (N)	
ypN0	12 (41%)
ypN1	4 (14%)
ypN2	2 (7%)
ypN3	11 (38%)
Distant metastasis (M)	
ypM0	23 (79%)
ypM1	6 (21%)*
Peritoneal metastasis (P)	
P0	29 (100%)
P1	0 (0%)
Peritoneal lavage cytology (CY)	
CYX	1 (3%)
CY0	27 (93%)
CY1	1 (3%)
ypStage	
0	5 (17%)
IA	2 (7%)
IB	3 (10%)
IIA	4 (14%)
IIB	2 (7%)
IIIA	0 (0%)
IIIB	6 (21%)
IIIC	1 (3%)
IV	6 (21%)
Residual tumor (R)	
R0	28 (97%)
R1	1 (3%)
Histological evaluation criteria of tumor response	
Grade 0	2 (7%)
Grade 1a	7 (24%)
Grade 1b	6 (21%)
Grade 2	8 (28%)
Grade 3	6 (21%)

^*^para-aortic lymph node 4, peritoneal lavage cytology 1, gallbladder 1. Pathological findings of gastric cancer are documented according to the Japanese Classification of Gastric Carcinoma, third English edition

### Long-term outcomes

**Fig. 2:**
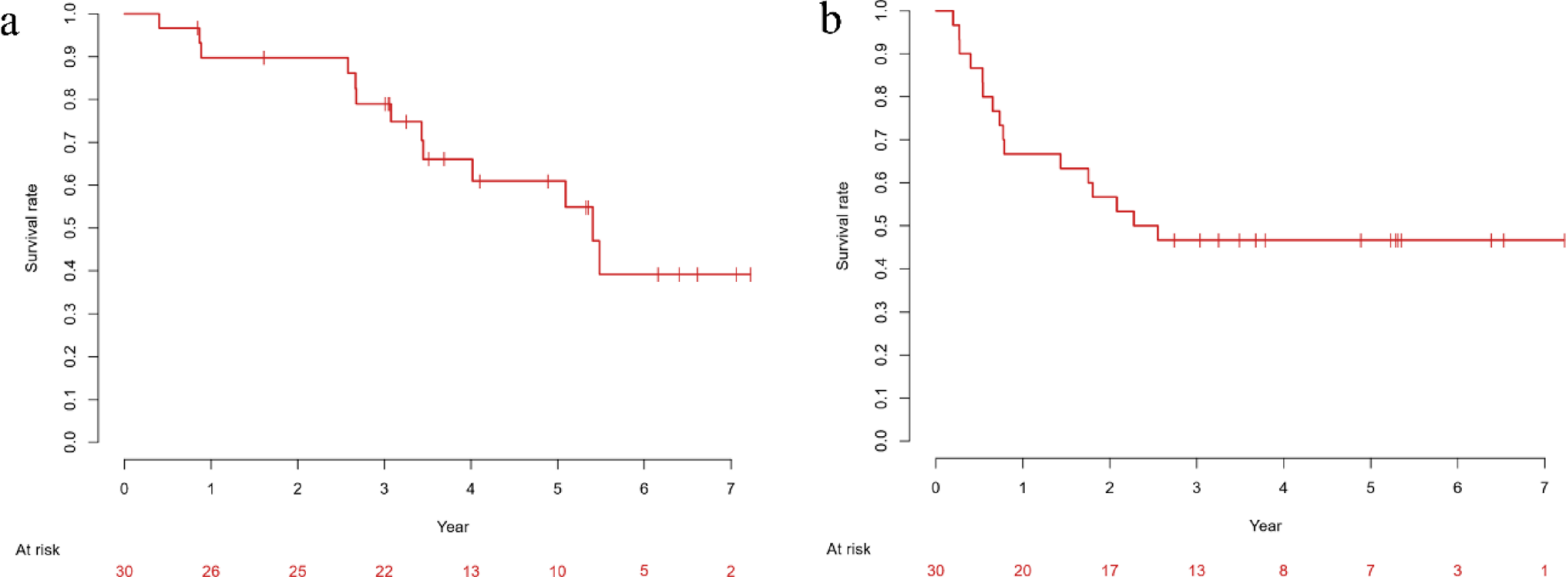
Kaplan–Meier estimates of overall survival (**a**) and progression-free survival (**b**). **a** The 3- and 5-year overall survival rate was 79.0% (95% CI 59.0–90.0), and 61.0% (95% CI 38.9–77.1), respectively. **b** The 3-year progression-free survival rate was 46.7% (95% CI 28.4–63.0).

As of the data cut-off (June 2025), the minimum follow-up among survivors was 36 months, and the median follow-up duration for all patients was 41.8 months (range, 4.8–86.7). During this period, 13 patients died and 15 developed recurrent disease. Recurrence sites included LNs in 7 patients; peritoneum in 7; liver in 3; and lung, bone, adrenal gland, ascites, and pleural effusion in 1 patient each (some with multiple sites). Salvage chemotherapy was administered to 14 patients and radiotherapy was administered to 2 patients, both of whom also received chemotherapy. The chemotherapy regimens are summarized in Table S1. The median OS was 64.9 months (95% CI 41.2–not estimable). The 3-year and 5-year OS rates were 79.0% (95% CI 59.0–90.0) and 61.0% (95% CI 38.9–77.1), respectively (Fig. 2a). The median PFS was 29.0 months (95% CI 9.4–not estimable), and the 3-year PFS rate was 46.7% (95% CI 28.4–63.0) (Fig. 2b).

### Exploratory subgroup analyses

The exploratory subgroup analyses of OS and PFS are shown in Tables 3 and 4, respectively. Overall, the OS was similar across the subgroups, with no statistically significant differences. For PFS, patients aged < 70 years had better outcomes than those aged ≥ 70 years, with 3-year PFS rates of 60.0% versus 20.0% (HR 0.35; 95% CI 0.13–0.96; *p* = 0.041). No other baseline characteristics were significantly associated with PFS. Multivariate Cox regression analysis incorporating baseline clinical factors was performed to explore predictors of survival. For OS, younger age (< 70 years) and good performance status (PS 0) showed borderline associations with better survival, whereas differentiated histology was the only factor independently associated with better outcomes (Table S2). No clear associations were observed for other baseline clinical variables. Regarding PFS, no clinical variables were identified as independent prognostic factors (Table S3). Although younger age, PS 0, and lower nodal burden (cN1) tended to correlate with longer PFS, these trends did not reach statistical significance.

**Table 3:** Subgroup analysis for overall survival

		N	MST (mo) [95%CIs]	3 yr survival [95CIs]	5 yr survival [95%CIs]	HR [95% CIs]	*p*
Sex	M	24	61.1 [36.9, NE]	78.6% [55.9%, 90.5%]	56.6% [32.3%, 75.0%]	3.225 [0.415, 25.053]	0.262
	F	6	–	83.3% [27.3%, 97.5%]	83.3% [27.3%, 97.5%]		
Age, year	< 70	20	–	79.4% [54.0%, 91.7%]	73.7% [47.8%, 88.2%]	0.419 [0.139, 1.268]	0.123
	≥ 70	10	41.4 [10.6, NE]	77.8% [36.5%, 93.9%]	31.1% [ 4.6%, 64.1%]		
PS	PS0	25	65.8 [41.4, NE]	79.5% [57.4%, 90.9%]	62.6% [38.0%, 79.7%]	0.504 [0.136, 1.868]	0.305
	PS1	5	50.9 [10.6, NE]	75.0% [12.8%, 96.1%]	50.0% [ 5.8%, 84.5%]		
Histological type	Differentiated	14	65.8 [41.4, NE]	100.0% [-]	76.9% [44.2%, 91.9%]	0.390 [0.123, 1.236]	0.109
	Undifferentiated	16	48.2 [30.9, NE]	58.4% [29.6%, 78.9%]	39.0% [ 8.4%, 69.9%]		
Main tumor location	UE	13	61.1 [30.9, NE]	69.2% [37.3%, 87.2%]	59.3% [27.5%, 81.0%]	1.386 [0.464, 4.139]	0.558
	ML	17	64.9 [36.9, NE]	87.4% [58.1%, 96.7%]	61.8% [30.2%, 82.4%]		
cT	cT2-3	17	64.9 [41.4, NE]	81.6% [53.0%, 93.7%]	66.7% [37.2%, 84.8%]	0.549 [0.179, 1.690]	0.296
	cT4a	13	65.8 [10.6, NE]	75.0% [40.8%, 91.2%]	53.6% [21.0%, 77.9%]		
cN	cN1	10	–	80.0% [40.9%, 94.6%]	68.6% [30.5%, 88.7%]	0.356 [0.096, 1.328]	0.124
	cN2-3	20	61.1 [36.9, 65.8]	78.5% [52.3%, 91.4%]	57.1% [29.7%, 77.2%]		
cM	cM0	13	65.8 [36.9, NE]	84.6% [51.2%, 95.9%]	66.6% [33.1%, 86.1%]	1.007 [0.335, 3.032]	0.989
	cM1	17	64.9 [30.9, NE]	74.8% [45.8%, 89.7%]	57.0% [26.9%, 78.6%]		

*PS* Eastern Cooperative Oncology Group Performance Status, *MST* Median survival time, *CI* Confidence interval, *HR* Hazard ratio, *NE* not estimable. Clinical findings of gastric cancer are documented according to the Japanese Classification of Gastric Carcinoma, third English edition

**Table 4:** Subgroup analysis for progression-free survival

		N	MST (mo) [95%CIs]	3 yr survival [95CIs]	HR [95% CIs]	*p*
Sex	M	24	26.2 [9.3, NE]	41.7% [22.2%, 60.1%]	1.719 [0.389, 7.590]	0.474
	F	6	–	66.7% [19.5%, 90.4%]		
Age, year	< 70	20	–	60.0% [35.7%, 77.6%]	0.354 [0.131, 0.959]	0.041
	≥ 70	10	19.1 [2.4, 25.0]	20.0% [ 3.1%, 47.5%]		
PS	PS0	25	–	52.0% [31.2%, 69.2%]	0.441 [0.141, 1.376]	0.158
	PS1	5	9.3 [2.4, NE]	20.0% [ 0.8%, 58.2%]		
Histological type	Differentiated	14	27.3 [8.8, NE]	50.0% [22.9%, 72.2%]	0.714 [0.266, 1.922]	0.505
	Undifferentiated	16	23.9 [4.8, NE]	43.8% [19.8%, 65.6%]		
Main tumor location	UE	13	17.2 [6.4, NE]	38.5% [14.1%, 62.8%]	1.729 [0.645, 4.635]	0.276
	ML	17	–	52.9% [27.6%, 73.0%]		
cT	cT2-3	17	–	52.9% [27.6%, 73.0%]	0.579 [0.217, 1.548]	0.276
	cT4a	13	21.0 [6.4, NE]	38.5% [14.1%, 62.8%]		
cN	cN1	10	–	70.0% [32.9%, 89.2%]	0.327 [0.093, 1.153]	0.082
	cN2-3	20	21.3 [6.5, NE]	35.0% [15.7%, 55.2%]		
cM	cM0	13	30.6 [7.8, NE]	46.2% [19.2%, 69.6%]	0.972 [0.362, 2.613]	0.955
	cM1	17	27.3 [6.4, NE]	47.1% [23.0%, 68.0%]		

*PS* Eastern Cooperative Oncology Group Performance Status, *MST* Median survival time, *CI* Confidence interval, *HR* Hazard ratio, *NE* not estimable. Clinical findings of gastric cancer are documented according to the Japanese Classification of Gastric Carcinoma, third English edition

### Associations between treatment response, postoperative therapy, and survival

**Fig. 3:**
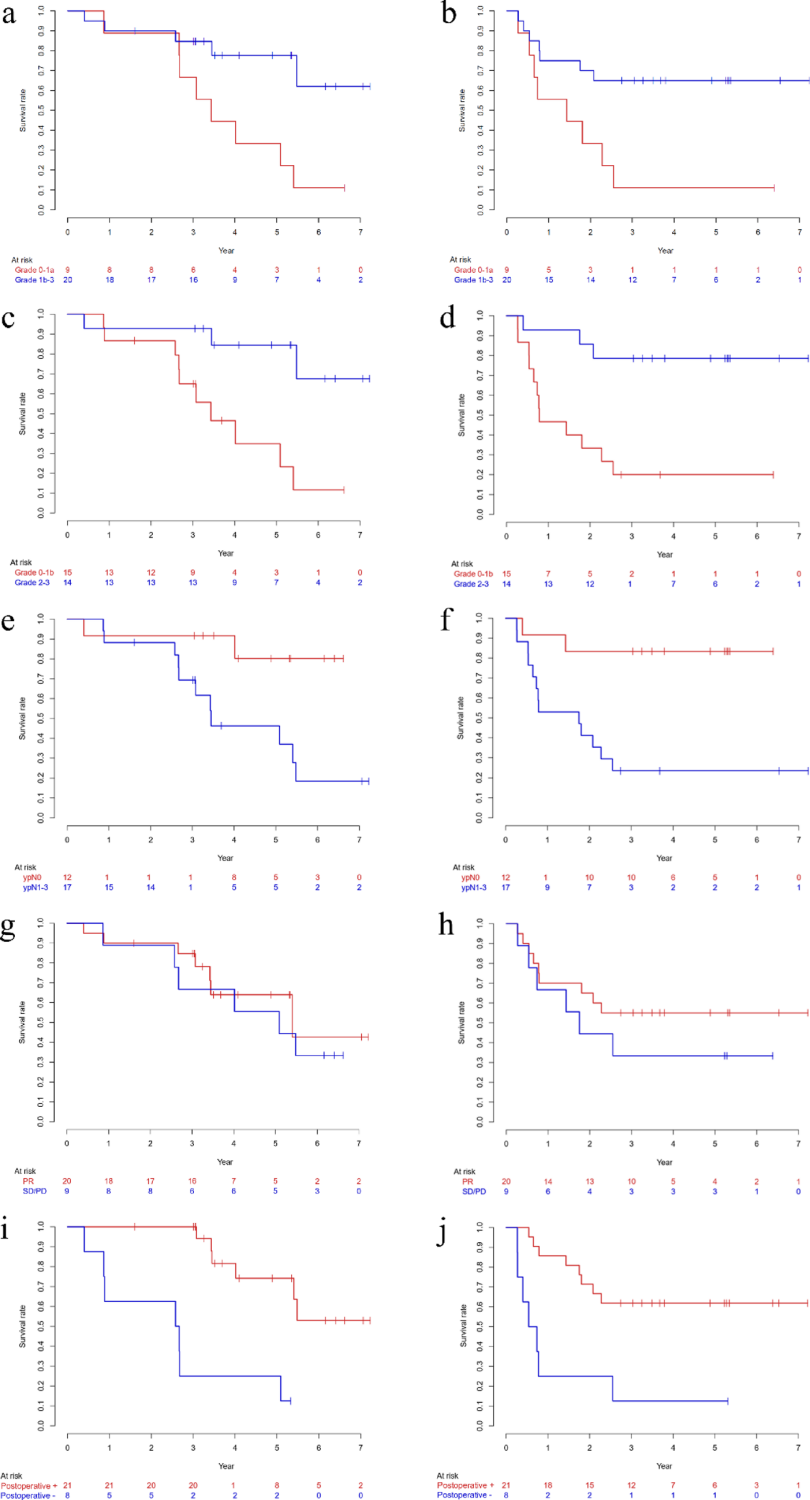
Kaplan–Meier estimates of overall survival (OS) curves and progression-free survival (PFS) curves by subgroups. OS (**a**, **c**) and PFS (**b**, **d**) by pathological response grade, OS (**e**) and PFS (**f**) by ypN, OS (**g**) and PFS (**h**) by response according to RECIST 1.0, and OS (**i**) and PFS (**j**) by postoperative chemotherapy

The pathological response grade was significantly correlated with survival. When patients were categorized into grade 0–1a versus ≥ 1b, those with a conventional pathological response (≥ 1b) had significantly better survival (OS: HR 0.255, 95% CI 0.083–0.784, *p* = 0.017; PFS: HR 0.278, 95% CI 0.100–0.774, *p* = 0.014) (Fig. 3a–b). This association became even more pronounced when pathological response was classified into grade 0–1b versus ≥ 2. Patients with a major pathological response (≥ 2) exhibited markedly better survival (OS: HR 0.173, 95% CI 0.046–0.649, *p* = 0.009; PFS: HR 0.160, 95% CI 0.045–0.575, *p* = 0.004) (Fig. 3c–d). The ypN0 status was also associated with better OS (HR 0.192, 95% CI 0.042–0.872, *p* = 0.032) and PFS (HR 0.144, 95% CI 0.032–0.642, *p* = 0.011) (Fig. 3e–f). In contrast, the radiologic response by RECIST (PR vs. SD/PD) demonstrated only a non-significant trend toward improved OS (HR 0.737, 95% CI 0.240–2.264, *p* = 0.594) and PFS (HR 0.583, 95% CI 0.207–1.643, *p* = 0.307) (Fig. 3g–h). Patients who received postoperative CapeOx had markedly better outcomes than those who did not (OS: HR 0.115, 95% CI 0.033–0.401, *p* < 0.001; PFS: HR 0.204, 95% CI 0.072–0.572, *p* = 0.002) (Fig. 3i–j).

## Discussion

The final survival analysis of the OGSG1701 trial demonstrated that perioperative CapeOx provides promising long-term outcomes in patients with gastric cancer and extensive LN metastases, a population traditionally associated with a poor prognosis. The regimen achieved a 66.7% response rate, a 93.3% R0 resection rate, and a pathological response (≥ grade 1b) rate of 66.7%. The median OS was 64.9 months, with 3- and 5-year OS rates of 79.0% and 61.0%. The median PFS was 29.0 months.

These results appear favorable when compared with previous Japanese phase II studies in similar high-risk populations. In JCOG0405, the R0 resection rate was 82%, with 3- and 5-year OS rates of 59% and 53%, respectively. In JCOG1002, the R0 resection rate was 83.0%, with 3- and 5-year OS rates of 62.7% and 54.9%, respectively. However, direct comparisons have been limited due to advances in systemic therapy over the past decade. In the OGSG1701, many patients received effective post-progression treatments such as ramucirumab plus (nab-)paclitaxel, nivolumab, or trifluridine/tipiracil, which likely contributed to improved survival. Nevertheless, the outcomes of our study support perioperative CapeOx as a viable strategy for patients with extensive LN metastasis.

Triplet regimens have also been investigated to improve efficacy in the preoperative setting. The phase II JCOG1704 trial of preoperative docetaxel, oxaliplatin, and S-1 (DOS) reported the highest pathological response rates among Japanese neoadjuvant regimens, including a 24% pathological complete response [14]. In Western countries, fluorouracil, leucovorin, oxaliplatin, and docetaxel (FLOT) have become standard preoperative regimens, achieving a 16% pathological complete response in the FLOT4 trial [15]. However, the toxicities of these triplet regimens may limit their applicability, particularly in elderly or frail patients. In this context, CapeOx offers a favorable balance among efficacy, tolerability, and practicality. The regimen requires no hydration and is administered in the outpatient setting, which is advantageous for Japan’s aging population with gastric cancer. The TCOG GI-1601 trial [16], which evaluated CapeOx in patients aged ≥ 70 years with advanced gastric cancer, demonstrated favorable feasibility and safety, supporting its applicability in older individuals. Nevertheless, whether perioperative CapeOx truly provides a favorable balance of efficacy and tolerability in elderly patients could not be definitively determined in OGSG1701. In exploratory subgroup analyses, patients aged ≥ 70 years showed significantly worse PFS and a numerically inferior OS. These findings should be interpreted cautiously, as the elderly subgroup consisted of only 10 patients and the results may partly reflect competing risks such as non–cancer-related mortality, which are more prevalent in older populations.

From another perspective, CapeOx represents the backbone of emerging multimodal strategies that combine immune checkpoint inhibitors, anti-HER2 therapies, and CLDN18.2-targeted agents. Thus, OGSG1701 provides timely bridging evidence for CapeOx-based perioperative combinations in patients with extensive nodal disease.

Compliance with postoperative chemotherapy is a major concern in patients who require extensive lymphadenectomy or multivisceral resection. In the OGSG1701 trial, 53% of the patients completed the entire protocol, including five postoperative cycles. This completion rate is acceptable in the high-risk populations and is consistent with the results of the OGSG1601 [17], which also used a perioperative CapeOx approach. Administering three cycles preoperatively may enhance systemic exposure and tumor downstaging while maintaining feasibility.

Exploratory analyses revealed that baseline clinical characteristics, including sex, performance status, tumor location, and clinical T/N factors did not significantly influence OS or PFS. In contrast, posttreatment pathological findings showed clear prognostic importance. Higher pathological response grades and ypN0 status were strongly associated with favorable outcomes, underscoring the relevance of tumor chemosensitivity and effective downstaging. Receipt of postoperative CapeOx was also associated with markedly better survival, although a selection bias must be considered. Interestingly, the RECIST v1.0–based radiologic response, which was used as the primary endpoint in line with previous phase II designs, did not correlate with OS or PFS. This highlights the limitations of RECIST in nodal-dominant disease, in which decreases in LN size may reflect fibrosis or inflammation rather than true tumor regression [18].

Moreover, pathological response provided clearer prognostic discrimination when a higher threshold of grade ≥ 2, rather than the conventional grade ≥ 1b, was applied. Although earlier JCOG studies such as JCOG0405 and JCOG1002 classified pathological responders as those achieving grade ≥ 1b, findings from the JCOG1004-A analysis indicate that stronger pathological response is more suitable as a surrogate for survival [19]. Consistent with this concept, the JCOG1704 trial, which employed the more intensive DOS regimen, adopted major pathological response defined as grade ≥ 2 as its primary endpoint. In this context, our findings support the notion that major pathological response, defined as grade ≥ 2, may represent a more robust surrogate endpoint for evaluating the effectiveness of perioperative chemotherapy in patients with extensive LN metastasis.

This study had several limitations. This was a single-arm trial with a modest sample size, which limits the statistical power and increases the risk of type II errors. Given the limited sample size and the small number of patients who completed protocol treatment, the subgroup and multivariable analyses (Tables 3, 4, S2, and S3) should be considered exploratory and hypothesis-generating rather than definitive. As eight patients were censored between 3 and 5 years after surgery, the estimated 5-year OS rate and median OS may be immature and potentially overestimated. Cross-trial comparisons require cautious interpretations because the therapeutic landscape for advanced gastric cancer has evolved substantially and modern post-progression therapies may have contributed to the favorable survival observed. Surgical procedures, pathological assessments, and perioperative management were performed locally without central standardization, potentially introducing inter-institutional variability. The radiologic response was assessed using RECIST v1.0, which has limited accuracy for nodal-dominant diseases. Finally, quality-of-life and functional outcomes were not evaluated.

Despite these limitations, the OGSG1701 trial provides prospective evidence supporting the feasibility and effectiveness of perioperative CapeOx in patients with gastric cancer and extensive LN metastases. These findings warrant evaluation in randomized phase III trials comparing perioperative doublet and triplet regimens, and in trials assessing their combination with immune checkpoint inhibitors, which are increasingly being incorporated into the therapeutic landscape for advanced gastric cancer.

## Conclusions

Perioperative CapeOx chemotherapy has achieved promising survival outcomes with acceptable toxicity in patients with advanced gastric cancer and extensive LN metastases. This regimen may be a feasible perioperative option and platform for future combination strategies.

## Supplementary Information

Below is the link to the electronic supplementary material.

Supplementary Material 1

## Declarations

### Human and animals rights

All procedures followed were in accordance with the ethical standards of the responsible committee on human experimentation (institutional and national) and with the Helsinki Declaration of 1964 and later versions. All patients provided written informed consent.
