# Supplementary material for: Long-term survival outcomes from a phase II trial of perioperative capecitabine plus oxaliplatin for advanced gastric cancer with extensive lymph node metastases: OGSG 1701
Source: Gastric Cancer. 2026 Mar 13;29(3):611–21. doi: 10.1007/s10120-026-01726-x (PMC13124816; doi:10.1007/s10120-026-01726-x)
Supplement: Supplementary file 1 — Supplementary Material 1 [file 10120_2026_1726_MOESM1_ESM.docx]

| Table S1. Salvage chemotherapy regimens | | |  |  |  |  |  |  |  |  |
| --- | --- | --- | --- | --- | --- | --- | --- | --- | --- | --- |
| 1st (N=14) | n (%) | 2nd (N=11) | n (%) | 3rd (N=9) | n (%) | 4th (N=6) | n (%) | 5th (N=3) | n (%) |  |
| RAM+(nab)PTX | 6 (42.9) | Nivo | 5 (45.5) | RAM+(nab)PTX | 3 (33.3) | FTD | 2 (33.3) | Nivo | 1 (33.3) |  |
| CapeOx | 2 (14.3) | S-1 | 2 (18.2) | CPT-11 | 3 (33.3) | Nivo | 2 (33.3) | RAM+PTX | 1 (33.3) |  |
| XPT | 1 (7.1) | XPT | 1 (9.1) | FTD | 3 (33.3) | CPT-11 | 1 (16.7) | SOX | 1 (33.3) |  |
| Capecitabine | 1 (7.1) | RAM+PTX | 1 (9.1) |  |  | Tmab | 1 (16.7) |  |  |  |
| SOX+Nivo | 1 (7.1) | nabPTX | 1 (9.1) |  |  |  |  |  |  |  |
| SOX | 1 (7.1) | RAM | 1 (9.1) |  |  |  |  |  |  |  |
| S-1 | 1 (7.1) |  |  |  |  |  |  |  |  |  |
| DS | 1 (7.1) |  |  |  |  |  |  |  |  |  |
| *RAM* Ramucirumab, *PTX* paclitaxel, *CapeOx* Capecitabine plus oxaliplatin, *XPT* Capecitabine plus cisplatin plus trastuzumab, *SOX* S-1 plus oxaliplatin, *Nivo* Nivolumab, *DS* Docetaxel plus S-1, *CPT-11* Irinotecan, *FTD* trifluridine-tipiracil, *Tmab* Trastuzumab | | | | | | | | | |  |
|  |  |  |  |  |  |  |  |  |  |  |

| Table S2. Multivariable analysis for overall survival | |  |  |  |  |
| --- | --- | --- | --- | --- | --- |
|  | Multivariate analysis | | Backward stepwise (AIC) | |  |
|  | HR [95%CIs] | *p* | HR [95%CIs] | *p* |  |
| Sex (male / female) | 1.950 [ 0.132, 28.779] | 0.626 | - |  |  |
| Age (＜70 y.o./ ≥70 y.o.) | 0.387 [ 0.096, 1.553] | 0.180 | 0.323 [ 0.101, 1.027] | 0.055 |  |
| PS (0 / 1) | 0.195 [ 0.033, 1.157] | 0.072 | 0.255 [ 0.060, 1.084] | 0.064 |  |
| Histological type (diff/ undiff) | 0.247 [ 0.057, 1.079] | 0.063 | 0.275 [ 0.080, 0.950] | 0.041 |  |
| Main tumor location (UE / ML) | 1.741 [ 0.455, 6.662] | 0.418 | - |  |  |
| cT (cT2-3 / cT4) | 1.729 [ 0.254, 11.751] | 0.575 | - |  |  |
| cN (cN1 / cN2-3) | 0.493 [ 0.081, 2.987] | 0.441 | - |  |  |
| cM (cM0 / cM1) | 1.689 [ 0.266, 10.717] | 0.578 | - |  |  |
| *PS* Eastern Cooperative Oncology Group Performance Status, *diff* differentiated. *undiff* undifferentiated, *HR* Hazard ratio, *CI* Confidence interval, *AIC* Akaike Information Criterion. Clinical findings of gastric cancer are documented according to the Japanese Classification of Gastric Carcinoma, third English edition. | | | | |  |
|  |  |  |  |  |  |
|  |  |  |  |  |  |

| Table S3. Multivariable analysis for progression-free survival | |  |  |  |  |
| --- | --- | --- | --- | --- | --- |
|  | Multivariate analysis | | Backward stepwise (AIC) | |  |
|  | HR [95%CIs] | *p* | HR [95%CIs] | *p* |  |
| Sex (male / female) | 0.646 [ 0.076, 5.467] | 0.688 | - |  |  |
| Age (＜70 y.o./ ≥70 y.o.) | 0.341 [ 0.113, 1.033] | 0.057 | 0.374 [ 0.134, 1.043] | 0.06 |  |
| PS (0 / 1) | 0.337 [ 0.075, 1.523] | 0.157 | 0.402 [ 0.125, 1.294] | 0.126 |  |
| Histological type (diff/ undiff) | 0.535 [ 0.168, 1.708] | 0.291 | - |  |  |
| Main tumor location (UE / ML) | 2.011 [ 0.634, 6.378] | 0.235 | - |  |  |
| cT (cT2-3 / cT4) | 1.912 [ 0.362, 10.096] | 0.445 | - |  |  |
| cN (cN1 / cN2-3) | 0.225 [ 0.033, 1.535] | 0.127 | 0.393 [ 0.109, 1.420] | 0.154 |  |
| cM (cM0 / cM1) | 1.834 [ 0.365, 9.205] | 0.461 | - |  |  |
| *PS* Eastern Cooperative Oncology Group Performance Status, *diff* differentiated. *undiff* undifferentiated, *HR* Hazard ratio, *CI* Confidence interval, *AIC* Akaike Information Criterion. Clinical findings of gastric cancer are documented according to the Japanese Classification of Gastric Carcinoma, third English edition. | | | | |  |
|  |  |  |  |  |  |
|  |  |  |  |  |  |
